# Reciprocal-space solvent flattening

**DOI:** 10.1107/S0907444999010033

**Published:** 1999-11-01

**Authors:** Thomas C. Terwilliger

**Affiliations:** a Structural Biology Group, Mail Stop M888, Los Alamos National Laboratory, Los Alamos, NM 87545, USA

**Keywords:** solvent flattening, reciprocal-space maximization, phase improvement

## Abstract

Solvent flattening is a powerful tool for improving crystallographic phases for macromolecular structures obtained at moderate resolution, but uncertainties in the optimal weighting of experimental phases and modified phases make it difficult to extract all the phase information possible. Solvent flattening is essentially an iterative method for maximizing a likelihood function which consists of (i) experimental phase information and (ii) information on the likelihood of various arrangements of electron density in a map, but the likelihood function is generally not explicitly defined. In this work, a procedure is described for reciprocal-space maximization of a likelihood function based on experimental phases and characteristics of the electron-density map. The procedure can readily be applied to phase improvement based on solvent flattening and can potentially incorporate information on a wide variety of other characteristics of the electron-density map.

## Abstract 2

A procedure is described for improvement of crystallographic phases by reciprocal-space maximization of a likelihood function including experimental phases and characteristics of the electron-density map.

## 1. Introduction

**Figure 1:**
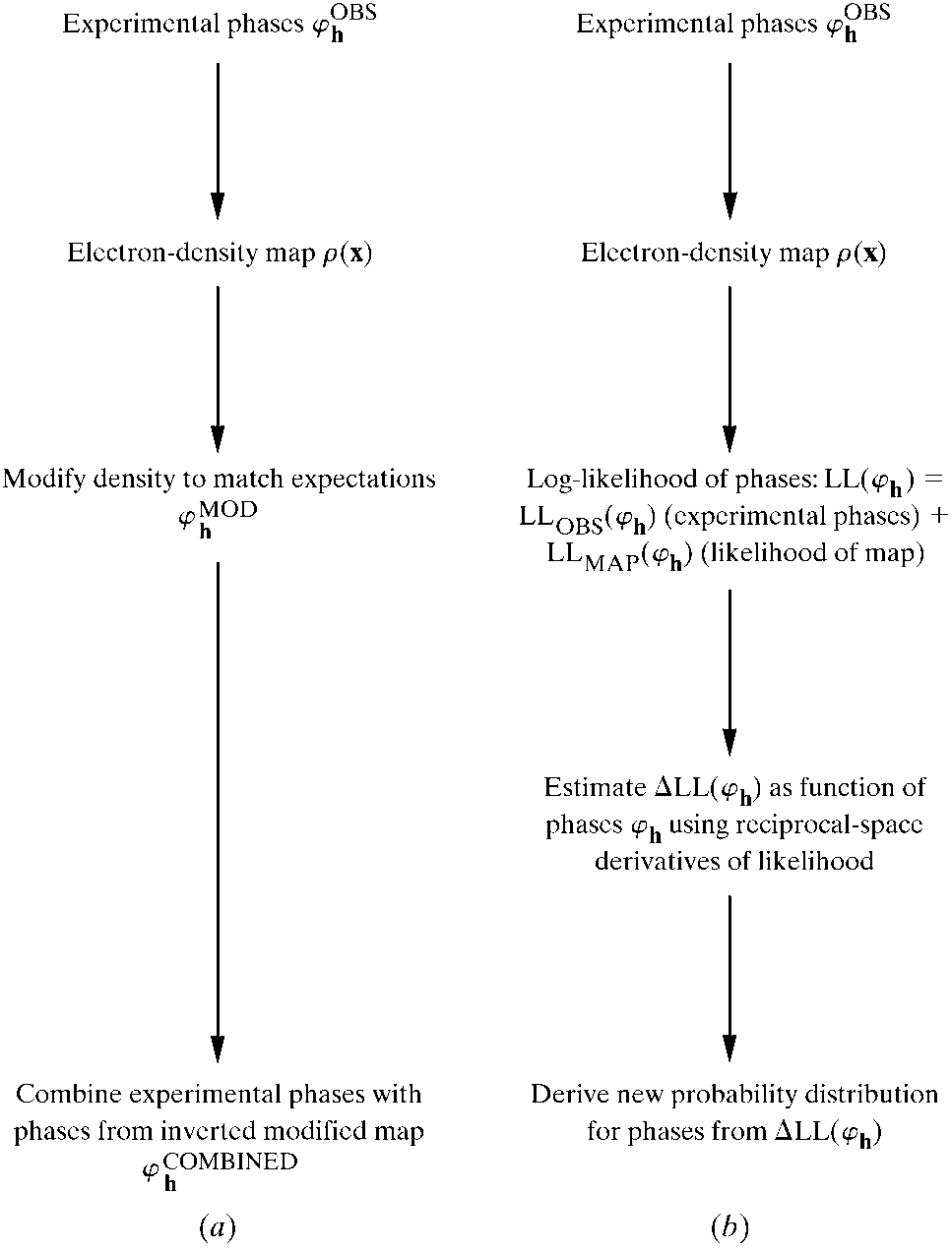
Flow diagrams for (*a*) real-space solvent flattening and (*b*) reciprocal-space solvent flattening.

Solvent flattening is one of the most powerful tools available for improving crystallographic phases for macromolecular structures obtained at moderate resolution (Wang, 1985 ▶). It is applied routinely, often in combination with other density-modification techniques such as non-crystallographic symmetry averaging, histogram matching, direct methods and entropy maximization (Abrahams & Leslie, 1996 ▶; Giacovazzo & Siliqi, 1997 ▶; Cowtan & Main, 1993 ▶, 1996 ▶; Goldstein & Zhang, 1998 ▶; Gu *et al.*, 1997 ▶; Lunin, 1993 ▶; Podjarny *et al.*, 1987 ▶; Prince *et al.*, 1988 ▶; Refaat *et al.*, 1996 ▶; Roberts & Brunger, 1995 ▶; Vellieux *et al.*, 1995 ▶; Xiang *et al.*, 1993 ▶; Zhang & Main, 1990 ▶; Zhang, 1993 ▶; Zhang *et al.*, 1997 ▶). The basis of solvent flattening is simple and elegant. Crystals of macromolecules often contain large contiguous solvent regions where the electron density is essentially constant. Consequently, a set of crystallographic phases which leads to a flat map in the solvent region is more likely to be correct than one which leads to high variation in the solvent. Application of a cycle of conventional real-space solvent flattening is straightforward (Fig. 1 ▶ *a*). The fraction of the unit cell made up by solvent can frequently be estimated from a knowledge of the contents of the unit cell, and the solvent regions can often be identified even in a poor electron-density map as the regions with lowest variation. A sharpened electron-density map [ρ(**x**)] based on experimental phases (ϕ_OBS_) is modified by flattening the solvent region. Modified crystallographic phases (ϕ_MOD_) are obtained from an inverse Fourier transform of the modified map. A probability distribution for these modified phases is then combined with one for the experimental phases to obtain a new set of phases (ϕ_COMBINED_). The new set of phases is consistent with the experimental data, but has a lower variation in the solvent region. Consequently, the new set of phases is generally a better estimate of the true phases than the experimental phases. This cycle of solvent flattening is typically repeated, recombining phases each time with the original experimental information, until the phases no longer change during a cycle. The improvement in the regions of the electron-density map which were not in the solvent region obtained by this process can be very substantial, particularly in cases where the fraction of solvent in the unit cell is greater than about 50%.

Although solvent flattening has been exceptionally useful, there are several reasons why its full potential has not been realised. One is that it is difficult to obtain an optimal weighting of the two sources of information in the critical step of combining experimental and modified phases (Roberts & Brunger, 1995 ▶; Cowtan & Main, 1996 ▶). This step is not straightforward because the probability distribution for the phases from the modified map (ϕ_MOD_) is not independent of the experimental phases (ϕ_OBS_). This can readily be appreciated in the case of an electron-density map with no solvent at all; in this case, the two distributions are identical and the phases from the modified map contribute nothing new and should be given zero weight. This difficulty has been partially side-stepped in several ways, including the use of maximum-entropy methods and the use of weighting optimized using cross-validation (Xiang *et al.*, 1993 ▶; Roberts & Brunger, 1995 ▶, Cowtan & Main, 1996 ▶) and ‘solvent flipping’ (Abrahams & Leslie, 1996 ▶).

A second reason solvent flattening has not realised its full potential is that although it produces a map with low variation in the solvent region yet consistent with experimental data, there is no assurance that this map is the one which best satisfies these criteria. Solvent flattening is essentially an iterative method for maximizing a likelihood function which consists of (i) experimental phase information and (ii) information on the likelihood of various arrangements of electron density in a map. Owing to the difficulties in weighting and the fact that the likelihood function is generally not precisely defined, however, it is difficult for conventional solvent-flattening procedures to make optimal use of this information.

In this work, a procedure is described for reciprocal-space maximization of a likelihood function based on experimental phases and characteristics of the electron-density map. This procedure can readily be applied to phase improvement based on solvent flattening and can potentially be extended to incorporate information on a wide variety of other characteristics of electron-density maps.

## 2. Reciprocal-space solvent flattening

Fig. 1 ▶(*b*) illustrates an approach to density modification based on reciprocal-space maximization of a likelihood function. The figure shows one cycle of solvent flattening. The overall procedure consists of calculating an electron-density map and determining a new probability distribution for the phase of each reflection based on experimental data and on the flatness of the solvent as a function of that phase. As in conventional solvent flattening, an electron-density map is calculated using experimentally determined structure factors. These amplitudes and phases have an *a priori* probability distribution [*P*_o_({*F* **_h_**, ϕ**_h_**})] based on the experimental data associated with them.

A log-likelihood function for a set of phases and amplitudes of structure factors LL({*F* **_h_**, ϕ**_h_**}) is then constructed by combining log-likelihood functions of two types: one [LL_OBS_({*F* **_h_**, ϕ**_h_**})] based on the experimentally derived probability distribution and the other [LL_MAP_({*F* **_h_**, ϕ**_h_**})] based on the characteristics of the electron-density map, 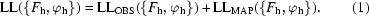
The map log-likelihood function LL_MAP_({*F* **_h_**, ϕ**_h_**}) (described in detail below) is designed to be a measure of the correspondence between the characteristics of the map and those expected of a macromolecular electron-density map. In the case of solvent flattening, it would ideally be the likelihood of the electron-density distribution in the solvent region.

To improve the quality of phasing for a map, a set of phases which increases the log-likelihood function needs to be found. One way to accomplish this is by calculating derivatives of the log-likelihood function with respect to structure factors in reciprocal space and using these derivatives to estimate new probability distributions for the phases. In our implementation, this process is greatly simplified by considering each phase independently of the others. In this case, the probability distribution for the phase of a particular reflection is proportional to the likelihood function calculated using that phase. This likelihood function can in turn be approximated using a Taylor series expansion based on the reciprocal-space derivatives of the log-likelihood function.

In essence, each cycle of reciprocal-space solvent flattening uses the observed phase information and the characteristics of the electron-density map to generate new estimates of a probability distribution for each phase. Although this does not directly maximize the likelihood function, the phases which are most probable according to this formulation are those which lead to the highest values of the likelihood function. The phases obtained from one cycle of solvent flattening can then be used to calculate a centroid electron-density map which is used in the next cycle.

## 3. Likelihood function for an electron-density map

The procedure outlined in Fig. 1 ▶(*b*) requires a readily calculable log-likelihood function for crystallographic phases based on the characteristics of the electron-density map. Additionally, the derivatives of the log-likelihood function are in the ideal case calculable in reciprocal space. (If they are not, then other standard methods of optimization which do not use derivatives could be used.)

A simple log-likelihood function with these properties was developed in several steps. If every grid point in an electron-density map were independent of every other point, the log likelihood of an arrangement of electron density in a map (LL_MAP_) would simply be the sum of the log likelihoods of the electron density {LL[ρ(**x**)]} at all points in the map. In a real map, neighboring points are not independent for a number of reasons, including the fact that the resolution of a map is finite. For example, a map calculated with only the *F*_000_ term has only one independent point in the unit cell. This effect can be taken into account in an approximate way by noting that the number of degrees of freedom in a map is roughly equal to the number of reflections used to calculate it. Another reason for a lack of independence of neighboring points in a map is that the likelihood of an arrangement of electron density could depend on patterns which involve a number of points in the map. For the present purpose, only likelihood functions which can be formulated in an approximate way involving only one point at a time will be considered. Using this approach, the overall log likelihood of an arrangement of electron density in an electron-density map calculated using *N*_REF_ independent reflections ({*F* **_h_**, ϕ**_h_**}) can be written approximately as
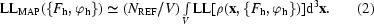
For the purpose of solvent flattening, an approximate expression for the log likelihood of the electron density at a particular location **x** in a map {LL[ρ(**x**, {*F* **_h_**, ϕ**_h_**})]} is needed which depends on whether the point **x** is within the solvent region or the protein region. One way to explicity incorporate information on the environment of **x** is to write the log-likelihood function LL[ρ(**x**, {*F* **_h_**, ϕ**_h_**})] as the log of the sum of conditional probabilities dependent on the environment of **x**: 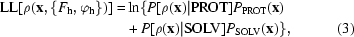
where *P*_PROT_(**x**) is the probability that **x** is in the protein region and *P*[ρ(**x**)|PROT] is the conditional probability for ρ(**x**) given that **x** is in the protein region; *P*_SOLV_(**x**) and *P*[ρ(**x**)|SOLV] are the corresponding quantities for the solvent region. As the identification of the solvent and protein regions involves an average over many grid points of the local variance of electron density (see below), the probability *P*_SOLV_(**x**) that a particular point is in the solvent region can be treated as if it were independent of the conditional probability *P*[ρ(**x**)|SOLV] for ρ(**x**) given that **x** is in the solvent region. In a more precise treatment, the correlation of these probabilities could also be considered, but this would make the analysis much more complicated.

For the limited purpose of solvent flattening, the probability distribution for the protein region {*P*[ρ(**x**)|PROT]} can be a constant, indicating that no information about the protein region is being used. In the solvent region, where the electron density is expected to be nearly constant, the probability distribution *P*[ρ(**x**)|SOLV] can be written simply as
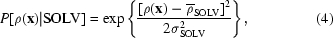
where 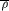
_SOLV_ is the mean value of the electron density in the solvent region and σ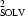 is its variance. The value of σ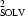 is non-zero even in a map calculated with perfect phases because the resolution of the reflection data would have to be infinite for the the electron density in the solvent region to be completely flat. Values of 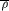
_SOLV_ and σ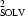 are estimated in our approach from the mean and standard deviation of the electron density in the solvent region at the beginning of a cycle of solvent flattening, but they could be estimated from a theor­etical analysis of the expected variance given the resolution of the data and also the model of the solvent envelope.

The probability *P*_SOLV_(**x**) that a particular location **x** is within the solvent region can be estimated from the local variation in electron density and a knowledge of the fraction of the unit cell in the solvent region (*f*_SOLV_; Wang, 1985 ▶; Leslie, 1987 ▶). In our approach, the probability that a particular point in the map is within the protein region is estimated in two steps. First, an approximate mask for the protein region is obtained with two iterations of a method very similar to that described by Wang (1985 ▶) as modified by Leslie (1987 ▶). The purpose of the mask is only to estimate the mean and standard deviation of electron density in the protein and solvent areas, not to explicitly delineate these regions. Our approach requires an estimate of the mean value of the electron density in the solvent region. In the first iteration, the mean value of the map in the solvent region is estimated from the mean value of the map overall. In the second iteration, the mask is used to estimate the mean value in the solvent region. A sharpened electron-density map is calculated using the current centroid phases, figures of merit and structure-factor amplitudes. The current estimate of the mean value of the map in the solvent region is then subtracted, values of the electron density are truncated at ±3σ and the map is squared. The resulting squared map is smoothed using a spherical cone function (Wang, 1985 ▶) with a radius *r*_Wang_ (typically 3–8 Å) to yield a squared smoothed map *Z*(**x**). The *f*_SOLV_ lowest portion of *Z*(**x**) is considered to be the solvent region. The value of the smoothing radius *r*_Wang_ is set by the empirical relation *r*_Wang_ (Å) = 7.5 (*d*_MIN_ 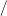
3 Å)(1/2〈*m*〉), where *d*_MIN_ is the high-resolution limit of the data and 〈*m*〉 is the mean figure of merit of the phasing.

The second step in estimating *P*_SOLV_(**x**) is based on the mean and standard deviation of the smoothed squared map *Z*(**x**) in the protein region (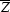
_PROT_ and σ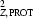, respectively) and in the solvent region (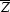
_SOLV_ and σ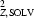, respectively). We use Bayes’ rule (Box & Tiao, 1973 ▶) to write that 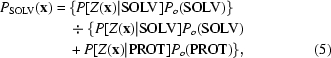
where *P*_*o*_(SOLV) = *f*_SOLV_ and *P_o_*(PROT) = 1 − *f*_SOLV_ are the *a priori* probabilities that **x** is in the solvent and protein regions, respectively. The probability distribution for the squared smoothed density *Z*(**x**) given that **x** is in solvent can be written as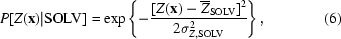 with an analogous relation holding for *P*[*Z*(**x**)|PROT].

## 4. Reciprocal-space derivatives of the log-likelihood of the map LL_MAP_({*F* **_h_**, ϕ**_h_**})

The log-likelihood function LL({*F*_**h**_, ϕ_**h**_}) in (1) could be maximized using any of a variety of procedures, but maxim­ization can be greatly facilitated by obtaining derivatives of the log-likelihood function for the electron-density map [LL_MAP_({*F*_**h**_, ϕ_**h**_})] with respect to the crystallographic structure factors. In this case, the overall log-likelihood function LL({*F*_**h**_, ϕ_**h**_}) can be approximated for all possible values of each phase (and amplitide if desired) and a probability distribution for each phase can be readily obtained.

In the approach described here, the first and second deriv­atives of the log likelihood of the map with respect to each structure factor are calculated, neglecting all cross-deriv­atives involving more than one structure factor. The calculation of derivatives is greatly simplified by neglecting correlations among structure factors, though this simplification can slow the convergence of maximization procedures and can affect the estimates of uncertainties associated with each phase.

The log-likelihood function for the electron-density map LL_MAP_({*F*_**h**_, ϕ_**h**_}) depends on the phases and amplitudes ({*F*_**h**_, ϕ_**h**_}) through both ρ(**x**) and, indirectly, through the probabilities that **x** is in solvent or protein [*P*_SOLV_(**x**) and *P*_PROT_(**x**)] (2). As mentioned above, the probability that **x** is in protein or solvent is generally much better defined than the value of the electron density ρ(**x**), and we ignore the contribution of *P*_SOLV_(**x**) and *P*_PROT_(**x**) to the derivatives of LL({*F*_**h**_, ϕ_**h**_}) in this analysis.

The derivatives of the log-likelihood function can be calculated with respect to any of several independent pairs of variables which represent the structure factor *F*_**h**_exp(*i*ϕ) and its possible changes, including *F*_**h**_ and ϕ_**h**_, its components *A*_**h**_ and *B*_**h**_ or, as we calculate here, with respect to changes in *F*_**h**_exp(*i*ϕ) along the directions of exp(*i*ϕ) and exp(*i*ϕ + *i*π/2) (*F*_**h**,∥_ and *F*_**h**,⊥_, respectively).

Differentiating (2) with respect to *F*_**h**,∥_ for a particular reflection indexed by **h** we obtain an expression for the first derivative of the map log-likelihood function, 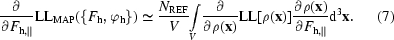
The electron density ρ(**x**) can be expressed as
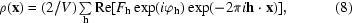
where one hemisphere of reflections is included and **h** = *h* a* + *k* b* + *l* c*. The derivative of ρ(**x**) with respect to *F*_**h**,∥_ for a particular index **h** is given by
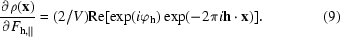
Substituting (9) into (7) and rearranging yields 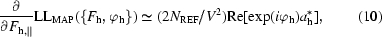
where the complex number *a*_**h**_ is simply a term in the Fourier transform of 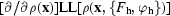,
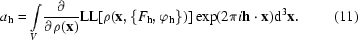
(10) can be generalized to include the effects of space-group symmetry, leading to the following expression for the first derivative of the map log-likelihood function with respect to *F*_**h**,∥_, 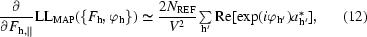
where the indices **h**′ are all indices equivalent to **h** owing to space-group symmetry. (10) to (12) emphasize that a Fast Fourier transform can be used to calculate the first derivative of any map log-likelihood function LL_MAP_({*F*_**h**_,ϕ_**h**_}) consisting of an integral of a log-likelihood function LL[ρ(*x*, {*F*_**h**_, ϕ_**h**_})] which can be differentiated with respect to ρ(**x**). This is very important for the speed of reciprocal-space solvent flattening.

To apply (10) and (12) to the case of solvent flattening, we use (3) {note the assumption that *P*[ρ(**x**)]|PROT] = 1} and take *P*[ρ(**x**)|SOLV] from (4) to write that 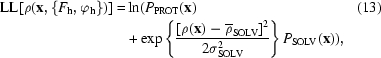
where *P*_PROT_(**x**) and *P*_SOLV_(**x**) are to be treated as constants in what follows. Then, differentiating (13) with respect to ρ(**x**), we obtain a simple expression for the derivative in the integrand of (11), 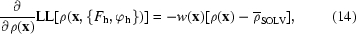
where the weighting factor *w*(**x**) is given by 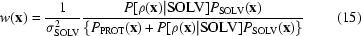
and *P*[ρ(**x**)|SOLV] is given in (4). Using (14) and (15), we are now in a position to evaluate (10) to (12).

The second derivative of the map log-likelihood function with respect to *F* **_h,∥_** can be obtained in a very similar fashion, but noting that *w*(**x**) depends on ρ(**x**) through (15), leading to the expression 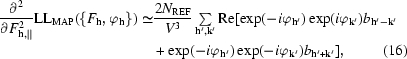
where the indices **h**′ and **k**′ are indices equivalent to **h** owing to space-group symmetry and where the coefficients *b*_**h**_ are again terms in a Fourier transform, 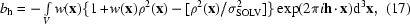
where *w*(**x**) was given in (15).

A very similar approach leads to expressions for the first and second derivatives of the map log-likelihood function with respect to changes in *F*_**h**_exp(*i*ϕ) along the direction of exp(*i*ϕ + *i*π/2): 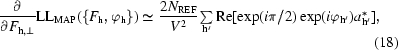

            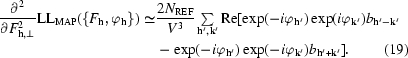

         

## 5. Probability distribution for phases including experimental information and characteristics of the map

Using (1) and (12) to (19), we can now write an approximate expression for the log likelihood of any value of a particular structure factor *F*_**h**_exp(iϕ_**h**_) using the first four terms of a Taylor series expansion around the value obtained with the starting structure factors {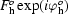} used in this cycle of density modification, combined with the prior log-likelihood LL_MAP_({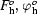}), 
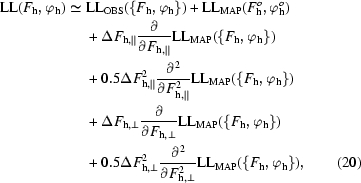
where Δ*F*_**h**,∥_ and Δ*F*_**h**,∥⊥_ are the differences between *F*_**h**_exp(iϕ_**h**_) and {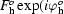)} along the directions of 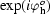 and 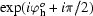, respectively.

For the case of solvent flattening, the amplitudes of structure factors are generally known quite precisely, while the phases are not. In this case, (20) can be readily used to calculate a new probability distribution for the reflection with indices **h**, 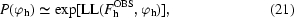
where 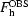 is the experimentally measured structure-factor amplitude. In other cases, such as phase extension, the amplitude of the structure factor may be unknown. In these cases, the expression in (20) can be calculated over all values of the magnitude and phase to obtain a two-dimensional probability distribution for the structure factor.

## 6. Implementation

(20) and (21) can be used to carry out one cycle of reciprocal-space density modification. As the expressions are not exact, the new phase-probability distributions obtained are only approximations. Iterations of several cycles of reciprocal-space density modification can be used to improve these estimates. Additionally, these iterations can be interspersed with cycles of estimation of the probability that each point in the map is in the solvent region (5). At the start of each cycle, the amplitudes and phases used (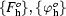) ordinarily correspond to those of a sharpened centroid electron-density map. The sharpening is performed by finding an overall temperature factor which optimizes the fit in shells of resolution of the observed structure factors to those of a model protein structure (Cowtan & Main, 1998 ▶). The temperature factor for the observed data used for sharpening is then taken to be the sum of that for the model data and the fitted temperature factor. As the solvent-flattening procedure described here only restricts density in the solvent to values near the mean in the solvent region, it is convenient to adjust the *F*_000_ term in the Fourier synthesis at the beginning of each cycle so that the mean electron density in the solvent region (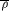
_SOLV_) is zero.

## 7. Comparison of real-space and reciprocal-space solvent flattening with model and real data

To evaluate the utility of reciprocal-space solvent flattening, it was applied to both model and real data and the results were compared with those obtained with real-space solvent flattening. To make the comparison as realistic as possible, an attempt was made to use the same information in both the real-space and reciprocal-space implementations. Consequently, no histogram matching, truncation or other real-space density modifications were applied in either case and the the real-space solvent flattening was performed using cross-validation to optimize the weighting of model and starting phases (Cowtan & Main, 1996 ▶).

**Figure 2:**
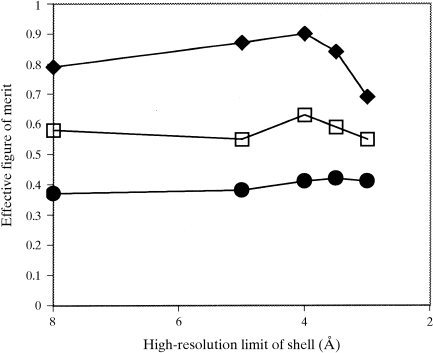
Correlation of solvent-flattened phases with true phases [〈cos(Δϕ)〉] for model data in a unit cell containing 70% solvent as a function of resolution. Structure factors (6906 model data from ∞ to 3.0 Å) were generated based on coordinates from a dehalogenase enzyme from *Rhodococcus* species ATCC 55388 (American Type Culture Collection, 1992 ▶) determined recently in our laboratory (J. Newman, personal communication), except that only the N-terminal 174 residues (of 267) were included in the calculation in order to simulate a unit cell with 70% solvent. The calculation was performed in space group *P*2_1_2_1_2 with unit-cell dimensions *a* = 94, *b* = 80, *c* = 43 Å and one molecule in the asymmetric unit. Electron density for the solvent region was introduced by calculating a model electron-density map based on protein atoms alone, setting the mean electron density in the solvent region (greater than 2.5 Å from any protein atom) to 0.32 e Å^−3^ and the mean electron density in the protein region to 0.43 e Å^−3^, respectively, smoothing the interface between solvent and protein region to minimize the introduction of high-frequency terms and calculating an inverse Fourier transform to obtain model phases and amplitudes. Phases with simulated errors were generated by adding phase errors with a distribution given by *P*(Δϕ) = exp[*A*cos(Δϕ) + *C*cos^2^(Δϕ)], with the values *A* = 0.8 and *C* = 0.4 for acentric reflections and *A* = 0.4 and *C* = 0.2 for centric reflections. This led to an average value of the cosine of the phase error (*i.e.* the true figure of merit of the phasing) of 〈cos(Δϕ)〉 = 0.42 for acentric and 0.39 for centric reflections. The model data with simulated errors was then solvent flattened by the reciprocal-space method as described here and by the real-space method as implemented in the program *dm* (Cowtan & Main, 1996 ▶), version 1.8, using solvent flattening and omit mode. Although *dm* will carry out solvent flattening alone in this way, it should be noted that this is a non-recommended mode (all recommended modes also contain histogram matching, which we did not include in order to keep the comparison restricted to the use of solvent flattening). Circles, starting phases; squares, real-space solvent flattening; diamonds, reciprocal-space solvent flattening.

Fig. 2 ▶ illustrates the quality of phases obtained after real-space and reciprocal-space solvent flattening of a set of phases constructed from a model with 30% of the volume of the unit cell taken up by the protein model. The initial effective figure of merit of the phases [〈cos(Δϕ)〉] was 0.40 overall. As anticipated based on the high solvent content of the unit cell, both real-space and reciprocal-space solvent flattening improved the quality of phasing considerably, but reciprocal-space solvent flattening produced phases with a mean value of the effective figure of merit [〈cos(Δϕ)〉] of 0.80, much higher than the value of 0.57 obtained with real-space solvent flattening. An improvement in phase quality was found for both low-resolution and high-resolution data with reciprocal-space solvent flattening, but the most substantial improvement was with low- and medium-resolution data, where the effective figure of merit of the reciprocal-space solvent-flattened map was as high as 0.90.

**Figure 3:**
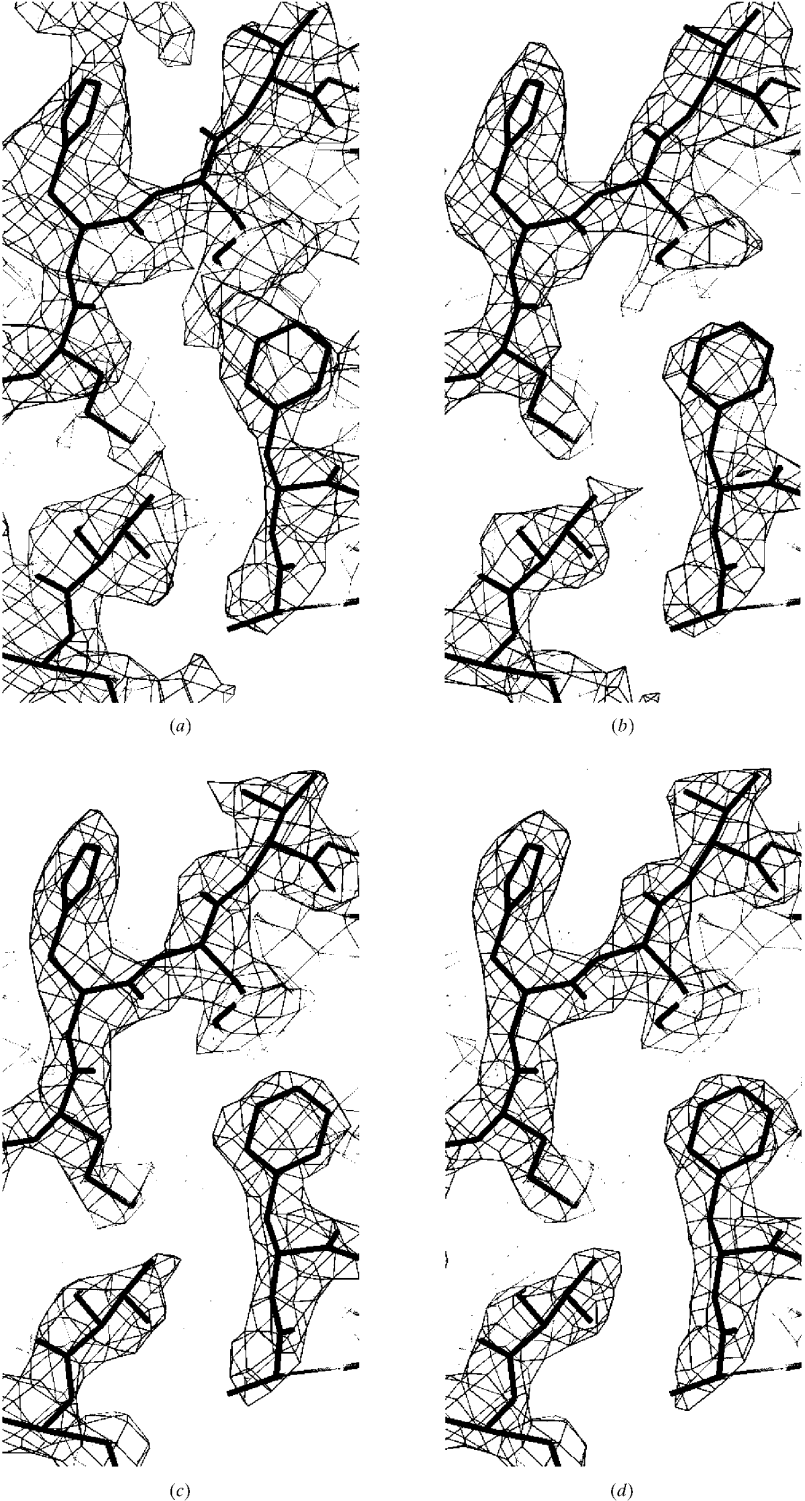
Sections of electron density in protein region of maps calculated as in Fig. 2 ▶ for the case with 70% solvent content. The maps shown are for (*a*) the starting phases, correlation coefficient to model map 0.42, (*b*) the real-space solvent-flattened phases, correlation coefficient 0.73, (*c*) the reciprocal-space solvent-flattened phases, correlation coefficient 0.93, and (*d*) model phases.

The quality of the flattened electron-density maps obtained using real-space and reciprocal-space solvent flattening are compared in Fig. 3 ▶. In this case with very high solvent content, the electron-density map obtained with reciprocal-space solvent flattening is much clearer than the one obtained with real-space solvent flattening. The correlation coefficient of the reciprocal-space solvent-flattened map to the model map is 0.93, while that of the real-space solvent-flattened map is 0.73.

**Figure 4:**
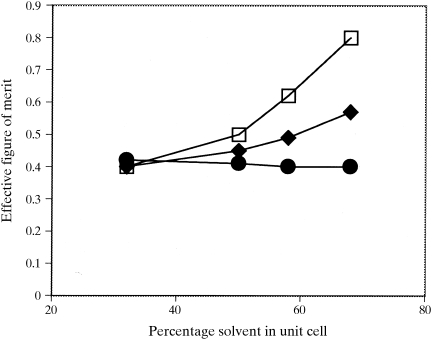
Correlation of solvent-flattened phases (diamonds, real space; squares, reciprocal space) with true phases (〈cos(Δϕ〉) for model data as in Fig. 2 as a function of the fraction of volume in the unit cell occupied by the solvent (see text).

As the phases used in Figs. 2 ▶ and 3 ▶ are calculated from a model, it is easy to investigate the effect of the fraction of solvent content in the unit cell on the utility of the solvent-flattening procedures. Fig. 4 ▶ shows the overall effective figure of merit of phasing [〈cos(Δϕ)〉] obtained using real-space and reciprocal-space solvent flattening on data calculated with protein models filling from 30 to 70% of the unit cell. In cases with very high solvent content, both approaches result in considerably improved phasing, with reciprocal-space solvent flattening yielding phases of much higher quality than real-space solvent flattening. At lower solvent content, the effectiveness of solvent flattening decreases considerably; neither method yields improvement from the starting effective figure of merit of 0.40 when the solvent content is near 30%.

The utilities of real-space and reciprocal-space solvent flattening were also compared using experimental multiwavelength (MAD) data on initiation factor 5A (IF-5A) recently determined in our laboratory (Peat *et al.*, 1998 ▶). This structure is in space group *I*4 with unit-cell dimensions *a* = 114, *b* = 114, *c* = 33 Å, one molecule in the asymmetric unit and a solvent content of about 60%. The experimental MAD phasing used to solve this structure was based on three Se atoms in the asymmetric unit; the phasing was carried out to a resolution of 2.2 Å. The effective figure of merit of phasing relative to the final refined model [〈cos(Δϕ)〉] was very high (0.58). Table 1 ▶ shows the effective figure of merit of phasing relative to the final refined model of the experimental phases and the real- and reciprocal-space solvent-flattened phases. Both methods improved the phasing considerably, with reciprocal-space solvent flattening improving it somewhat more than the real-space version. In order to simulate cases where the phasing is not as good as this, two additional tests were carried out in which just one or two of the selenium sites were used in phasing. Table 1 ▶ shows that reciprocal-space solvent flattening was an improvement over real-space solvent flattening in all three cases.

## 8. Discussion

Reciprocal-space solvent flattening differs from real-space solvent flattening (Wang, 1985 ▶) in two significant ways. One is that the reciprocal-space formulation involves the maximization of an explicitly defined likelihood function and the second is that the ‘flattening’ of the solvent region is carried out in reciprocal space. The maximization of a likelihood function is important because the issue of weighting the prior phase information relative to the information from the modified map is automatically dealt with. In the reciprocal-space formulation, only new information on the characteristics of the map, not a repetition of the starting phase information, is brought in by considering the map likelihood. The calculation of reciprocal-space derivatives is important because it means that the likelihood function can be directly optimized with respect to the parameters (phases, amplitudes) which are available, rather than indirectly through a weighted combination of starting parameters with those derived from flattened maps. Additionally, calculation of reciprocal-space derivatives by Fourier transform methods [(10) and (12)] can be carried out very quickly.

The idea of applying maximization of likelihood functions to improvement of crystallographic phases has been developed extensively by Bricogne and others (*e.g.*, Bricogne, 1984 ▶, 1988 ▶; Lunin, 1993 ▶), and it is generally recognized that density modification can in principle be carried out in either real or reciprocal space (Main, 1990 ▶). However, up to now the application of solvent flattening has always been carried out in real space, with any combination with the experimental phases carried out in reciprocal space and requiring a weighting scheme for combining the experimental and modified phases (Abrahams & Leslie, 1996 ▶; Cowtan & Main, 1993 ▶, 1996 ▶; Giacovazzo & Siliqi, 1997 ▶; Gu *et al.*, 1997 ▶; Lunin, 1993 ▶; Prince *et al.*, 1988 ▶; Roberts & Brunger, 1995 ▶; Vellieux *et al.*, 1995 ▶; Xiang *et al.*, 1993 ▶; Zhang & Main, 1990 ▶). The present methods demonstrate a simple approach to solvent flattening in reciprocal space using the maximization of a likelihood function. The approach developed here for reciprocal-space solvent flattening can also readily be extended to other types of density modification. The main restriction is that to apply our methods the derivatives of the likelihood function for the map with respect to electron density (or at least estimates of these derivatives) must be readily calculable. This allows (10) and (12) and related equations to be used. Most standard density-modification procedures (Podjarny *et al.*, 1987 ▶) can be formulated in this way. Density-modification procedures which are particularly well suited to our approach include non-crystallographic symmetry averaging (Rossmann & Blow, 1963 ▶), histogram matching using matching of moments (Goldstein & Zhang, 1998 ▶; Refaat *et al.*, 1996 ▶; Zhang & Main, 1990 ▶; Lunin, 1993 ▶), density truncation (Schevitz *et al.*, 1981 ▶), maximization of the distinction between protein and solvent regions (Terwilliger & Berendzen, 1999 ▶ *a*) and maximization of the connectivity of the electron-density map (Baker *et al.*, 1993 ▶).

## 9. Conclusions

The theory of reciprocal-space solvent flattening leads to an improved foundation for solvent flattening through the introduction of an explicit likelihood function which is maximized. This approach leads to improvements in the quality of crystallographic phases compared with those from real-space solvent flattening, which has required a relative weighting of model and starting phases that is difficult to carry out in an optimal fashion. The simplicity of reciprocal-space solvent flattening and its implementation with Fourier transform-based calculations of reciprocal-space derivatives of the likelihood function make it well suited for extension into other density-modification procedures.

## Figures and Tables

**Table 1:** Effective figure of merit of IF-5A solvent-flattened phases Mean values of the effective figure of merit [〈cos(Δϕ)〉] of solvent-flattened phases obtained with real-space or reciprocal-space solvent flattening relative to phases from the refined model of IF-5A (Peat *et al.*, 1998 ▶) are listed. Three sets of starting points were used, corresponding to inclusion of one to three of the Se atoms in the phasing model. Starting phases were calculated with *SOLVE* (Terwilliger & Berendzen, 1999 ▶ *b*).

Number of Se atoms included in phasing	Experimental	Real-space solvent flattening	Reciprocal-space solvent flattening
1	0.28	0.47	0.53
2	0.46	0.64	0.68
3	0.58	0.69	0.70
